# Comparison of Orthodontically Induced External Apical Root Resorption in Endodontically Treated and Vital Teeth: A Retrospective Split-Mouth Study

**DOI:** 10.7759/cureus.102287

**Published:** 2026-01-25

**Authors:** Arun Deepak, Bazila Malik, Jasleen Kour, Saraswati Raghunath Kokate, Sayali Jain, Shahinwaz Mulani, Seema Gupta, Rahul VC Tiwari

**Affiliations:** 1 Department of Orthodontics, RVS Dental College and Hospital, Coimbatore, IND; 2 Department of Conservative Dentistry and Endodontics, I.T.S. Dental College, Muradnagar, IND; 3 Department of Dentistry, Shri Mata Vaishno Devi Institute of Medical Excellence, Katra, IND; 4 Department of Orthodontics, Dr. Hedgewar Smruti Rugna Seva Mandal's Dental College and Hospital, Hingoli, IND; 5 Department of Orthodontics, Jawahar Medical Foundation's Annasaheb Chudaman Patil Memorial Dental College, Dhule, IND; 6 Department of Prosthodontics, Guru Gobind Singh College of Dental Science and Research Centre, Burhanpur, IND; 7 Department of Orthodontics, Kothiwal Dental College and Research Centre, Moradabad, IND; 8 Department of Oral and Maxillofacial Surgery, RKDF Dental College and Research Centre, Bhopal, IND

**Keywords:** endodontically treated teeth, orthodontic tooth movement, pulp, radiographs, root resorption

## Abstract

Background: Orthodontic tooth movement can induce biological responses in dental and periodontal tissues, most notably external apical root resorption (EARR). The behavior of endodontically treated teeth (ETT) during orthodontic therapy remains a subject of clinical interest, with conflicting evidence regarding their susceptibility to root resorption compared with vital teeth. This study aimed to compare the incidence and severity of orthodontically induced EARR in ETT and adjacent vital teeth and to identify factors associated with root resorption in both groups.

Materials and methods: This retrospective split-mouth cohort study evaluated the records of patients aged 18-30 years who underwent comprehensive fixed orthodontic treatment and had at least one endodontically treated tooth. ETT were compared with the adjacent vital teeth in the same patient. Pre- and post-treatment radiographs were analyzed to measure root length changes, and the resorption severity was categorized as none, mild, moderate, or severe. Demographic variables, tooth type, treatment duration, and pre-treatment root length were also recorded. Paired sample t-tests and chi-square tests were used for intergroup comparisons, and multivariable linear regression analysis was performed separately for endodontically treated and vital teeth to identify predictors of root resorption. Statistical significance was set at p < 0.05.

Results: Fifty patients were included, with a mean age of 25.66 ± 2.99 years and a mean treatment duration of 18.62 ± 4.72 months. The mean root resorption was significantly lower in ETT (0.34 ± 0.48 mm) than in vital teeth (1.15 ± 0.65 mm). Vital teeth exhibited a higher frequency of moderate and severe resorption, whereas most ETT showed no or mild resorption. Regression analysis revealed that treatment duration was the only significant predictor of root resorption in ETT, whereas both the treatment duration and pre-treatment root length were significant predictors in vital teeth.

Conclusion: ETT demonstrated significantly less orthodontically induced EARR than vital teeth. Treatment duration plays a key role in root resorption for both tooth types, suggesting that careful force control and treatment timing are essential to minimize adverse effects.

## Introduction

Orthodontic treatment plays a pivotal role in correcting malocclusions, enhancing esthetics, and improving oral function. However, the application of mechanical forces during tooth movement can induce biological responses in dental tissues, including the pulp and periodontal structures [1]. In vital teeth, orthodontic tooth movement (OTM) often leads to transient pulpal changes, such as hyperemia, reduced blood flow, and inflammatory reactions, which are generally reversible with controlled forces [2]. These alterations can manifest as increased enzymatic activity, apoptosis, and necrosis of the pulp cells, potentially culminating in pulpitis or necrosis if excessive or continuous forces are applied [3]. Additionally, OTM is associated with external apical root resorption (EARR), a common iatrogenic effect resulting from compression and inflammation in the periodontal ligament, with incidence rates varying based on force magnitude, duration, and individual factors, such as age and genetics [4]. While most resorption cases are minor and self-limiting, severe cases can compromise tooth longevity.

Endodontically treated teeth (ETT) that have undergone root canal therapy (RCT) due to pulp necrosis, infection, or trauma present unique challenges in orthodontic management. Since the pulp is removed during endodontic treatment, pulpal vitality is inherently absent, shifting the focus to the integrity of the periapical tissues and root structure [5]. The literature indicates that OTM on ETT can proceed safely if the endodontic treatment is of high quality, with proper cleaning, shaping, and obturation, minimizing risks [6]. Studies suggest that well-obturated ETT exhibit minimal apical remodeling or resorption during OTM, with no significant difference in EARR severity compared to vital teeth or even reduced EARR [7,8]. However, conflicting evidence exists; some reports highlight an increased susceptibility to inflammatory root resorption or ankylosis, particularly in ETT with prior trauma or incomplete apex formation [9]. Factors such as force type (intrusive forces), treatment duration, and pre-existing periapical pathology may exacerbate these effects. Despite these insights, the long-term impact of OTM on ETT remains understudied, with methodological limitations in existing research, including small sample sizes, underscoring the need for comprehensive evaluations. This retrospective study aimed to investigate the effects of orthodontic tooth movement on pulpal vitality and root resorption in ETT. The primary objective of this study was to compare the incidence and severity of orthodontically induced EARR between ETT and adjacent vital teeth. The secondary objective was to identify clinical and treatment-related factors associated with EARR in both endodontically treated and vital teeth.

## Materials and methods

Study design

This investigation was designed as a retrospective cohort study utilizing anonymized patient records from the Department of Orthodontics, Kothiwal Dental College and Research Centre, Moradabad, India, covering treatments completed between January 2015 and December 2024. Ethical approval was obtained from the Institutional Ethical Review Board (KDCRC/IERB/2/2025/SS08) prior to data extraction to ensure patient confidentiality. This study adhered to the STROBE (Strengthening the Reporting of Observational Studies in Epidemiology) guidelines for reporting retrospective studies [10].

Sample selection

The patient inclusion criteria encompassed individuals aged 18-30 years who underwent comprehensive orthodontic treatment involving fixed appliances and had at least one ETT prior to or during orthodontics. ETT were defined as teeth with documented RCT confirmed by pre-treatment radiographs and clinical records. Adjacent vital teeth served as intra-patient controls for comparison purposes. The exclusion criteria were as follows: systemic conditions affecting bone metabolism (such as osteoporosis and hyperparathyroidism), history of bisphosphonate use, incomplete orthodontic records, and ETT with poor-quality obturation (such as voids or overfills evident on radiographs). Additionally, teeth with pre-existing severe root resorption, ankylosis, or unresolved periapical pathology post-endodontics were excluded.

The sample size was calculated a priori using G*Power software (version 3.1.9.2, Heinrich Heine University, Düsseldorf, Germany). Based on a paired-comparison design (split-mouth), an effect size of 0.72 was adopted from a comparable study investigating root resorption in ETT versus vital teeth [11]. With an alpha level set at 0.05 and a desired power of 80%, the analysis indicated the requirement of 50 patients. This provided 50 paired observations (vital and treated teeth per patient), which was deemed sufficient to detect the specified effect. A total of 150 patients were screened, resulting in a sample of 50 ETT and 50 matched vital control teeth, selected via stratified random sampling to balance demographics such as age, sex, and malocclusion type (Class I, II, III).

Data collection methods

Data were extracted from electronic health records, including pre-orthodontic, mid-treatment, and post-orthodontic data. This comprised digital radiographs (periapical and panoramic), cone-beam computed tomography (CBCT) scans where available, clinical notes on vitality testing (electric pulp testing and cold tests), treatment plans detailing force magnitudes (such as 200 g via archwires or elastics), and durations (mean 18-24 months). Two calibrated investigators independently reviewed the records to ensure inter-rater reliability (kappa >0.8). Discrepancies were resolved by consensus with a senior orthodontist. Data were anonymized and stored in a secure database using software for structured entry, categorizing variables such as tooth type (incisors, premolars, molars), endodontic etiology (caries, trauma), and orthodontic mechanics (intrusion, extrusion, tipping).

Assessment of root resorption

EARR was quantified using pre- and post-treatment radiographs digitized and analyzed using the ImageJ software by two blinded examiners from different colleges (Arun Deepak and Jasleen Kour) to avoid examiner bias. Root length was measured from the cementoenamel junction to the apex along the long axis, with resorption calculated as the difference in root length (pre-treatment minus post-treatment) in mm. Resorption severity was classified based on the amount of length loss as follows: none (0 mm), mild (<2 mm), moderate (2-4 mm), or severe (>4 mm). Incidence was defined as any detectable resorption post-OTM (length loss >0 mm), while severity was compared between ETT and vital controls. Incidence was defined as any detectable resorption post-OTM, and severity was compared between ETT and vital controls. Risk factors, including force type and duration, were correlated with the resorption outcomes. Intra- and inter-examiner reliability were assessed using intraclass correlation coefficients (ICC >0.9).

Statistical analysis

Statistical analyses were performed by a blinded statistician (Shahinwaz Mulani) using IBM SPSS Statistics for Windows, Version 23 (Released 2015; IBM Corp., Armonk, New York, United States). Categorical variables are presented as frequencies and percentages. The distribution of continuous data was assessed using the Shapiro-Wilk test, with normally distributed data summarized as mean and standard deviation (SD). An independent samples t-test was used to compare the extent of root resorption between vital teeth and ETT. The association between the treatment group (vital vs. ETT) and the categorical severity of resorption was analyzed using the chi-square test. A multivariable regression analysis was conducted to identify significant predictors of resorption. A p-value of less than 0.05 was considered statistically significant for all the tests.

## Results

The study population comprised 50 patients with a mean age of 25.66 ± 2.99 years, and the average duration of orthodontic treatment was 18.62 ± 4.72 months. The cohort included 28 (56%) female patients and 22 (44%) male patients. The distribution of the analyzed teeth was as follows: 30 (60%) molars, 13 (26%) incisors, and 7 (14%) premolars (Table 1).

**Table 1:** Demographic and clinical characteristics of the study population. Continuous variables (age and duration) are presented as mean and standard deviation (SD), whereas categorical variables (sex and tooth type) are presented as frequency (n) and percentage (%). Percentages are calculated based on the total sample size (n = 50 patients; 50 paired teeth).

Parameter	Category	Value
Age (years)	Mean ± SD	25.66 ± 2.99
Duration of orthodontic treatment (months)	Mean ± SD	18.62 ± 4.72
Sex n (%)	Female	28 (56%)
	Male	22 (44%)
Tooth type, n (%)	Incisor	13 (26%)
	Premolar	7 (14%)
	Molar	30 (60%)

The paired t-test revealed a statistically significant difference in the extent of root resorption between the ETT and vital teeth (mean difference = -0.81 mm, p = 0.001). The mean resorption was markedly greater in vital teeth (1.15 ± 0.65 mm) than in ETT (0.34 ± 0.48 mm). The large Cohen's d effect size of 1.01 confirmed substantial clinical significance. This result indicates that vital teeth are significantly more susceptible to orthodontically induced EARR than teeth that have undergone prior endodontic treatment within the same patients (Table 2).

**Table 2:** Comparison of orthodontically induced root resorption between endodontically treated and vital teeth (paired analysis). Cohen’s d represents effect size; *p = 0.001 denotes a statistically significant value using the paired samples t-test.

Group	n	Mean root resorption (mm)	Std. Deviation	Mean difference (mm)	t value	p-value	Cohen's d
Endodontically treated teeth (ETT)	50	0.34	0.48	-0.81	-7.13	0.001*	1.01
Vital teeth	50	1.15	0.65				

The analysis revealed a statistically significant association between tooth type and root resorption severity (χ²=19.9, p=0.001). The ETT group demonstrated a more favorable outcome, with 24% of teeth exhibiting no resorption and 58% showing only mild resorption. In contrast, vital teeth were more susceptible to advanced resorption, with 23 (46%) and 5 (10%) teeth classified as having moderate and severe resorption, respectively. This distribution indicates that ETT exerts a protective effect against the severity of orthodontically induced inflammatory root resorption compared with vital teeth (Table 3).

**Table 3:** Severity distribution of external apical root resorption in endodontically treated and vital teeth. Percentages are calculated within each group; *p = 0.001 denotes a statistically significant value using the chi-square test.

Severity of resorption	Vital teeth n (%)	Endodontically treated teeth n (%)	χ² value	p-value
None (0 mm)	2 (4%)	12 (24%)	19.9	0.001*
Mild (< 2 mm)	20 (40%)	29 (58%)		
Moderate (2–4 mm)	23 (46%)	9 (18%)		
Severe (> 4 mm)	5 (10%)	0 (0%)		

Multivariate regression analysis revealed distinct predictors of root resorption in ETT and vital teeth. For ETT, only the duration of orthodontic treatment was significantly positively associated with root resorption (B=0.03, p=0.006). In contrast, for vital teeth, two factors were significant: treatment duration showed a strong positive association (B=0.13, p=0.001), whereas greater pre-treatment root length was a protective factor with a significant negative association (B=-0.4, p=0.001). Age and tooth type (molar/premolar) were not significant predictors in either of the models. These results indicate that the mechanisms influencing resorption differ between tooth types. Mechanical factors, represented by treatment time, are critical for ETT teeth, whereas for vital teeth, biological factors related to initial root morphology also play a substantial protective role, alongside treatment duration (Table 4).

**Table 4:** Multivariable linear regression analysis identifying predictors of root resorption. Regression coefficients (B) represent change in root resorption (mm) per unit increase in predictor; *p < 0.05 denotes statistically significant values.

Parameter	Endodontically treated teeth				Vital teeth			
	Coefficient (B)	t value	p-value	95% Confidence Interval	Coefficient (B)	t value	p-value	95% Confidence Interval
Age	0.02	0.69	0.496	-0.03 to 0.07	0	0.39	0.697	-0.02 to 0.03
Molar	0.12	0.74	0.462	-0.21 to 0.46	-0.06	-0.74	0.461	-0.21 to 0.10
Premolar	-0.19	-0.82	0.416	-0.65 to 0.27	0.01	0.1	0.921	-0.20 to 0.22
Duration of treatment	0.03	2.52	0.006*	0.02 to 0.04	0.13	17.94	0.001*	0.11 to 0.14
Pre-treatment root length	-0.06	-0.64	0.624	-0.25 to 0.13	-0.4	-1.42	0.001*	-0.80 to -0.10

## Discussion

The present retrospective cohort study demonstrated that ETT exhibited significantly less EARR during OTM than matched vital control teeth. Furthermore, the ETT exhibited a more favorable severity profile. These findings indicate that well-obturated ETT are more resistant to orthodontically induced EARR than vital pulp teeth, consistent with the majority of contemporary evidence. Grissom et al. [12] reported a greater average change in the surface area after OTM in vital teeth than in ETT.

The reduced EARR in ETT can be primarily attributed to the absence of vital pulp tissue, which eliminates inflammatory pulpal responses, neuropeptide release, and cytokine-mediated odontoclastic activation, which amplify apical remodeling in vital teeth under orthodontic forces. In vital teeth, mechanical stress triggers transient pulpal hyperemia, reduces blood flow, and initiates inflammatory cascades, promoting progressive inflammatory resorption at the apex. In contrast, devitalized ETT undergo predominantly superficial cemental or mechanical remodeling without biological amplification. This protective mechanism is strongly supported by recent systematic reviews and meta-analyses. Zhao et al. [13], in a comprehensive systematic review and meta-analysis, concluded that the EARR rate in ETT was significantly lower than in vital teeth, recommending priority endodontic therapy before orthodontics in combined cases to minimize resorption risks. Similarly, earlier meta-analyses reported mean differences of -0.48 mm and -0.45 mm favoring less resorption in ETT, with consistent evidence that endodontic treatment does not increase and may even reduce EARR susceptibility [14].

The significant association between tooth type and resorption severity, with vital incisors and premolars being more affected, reflects greater apical displacement, torque, and force concentration on anterior teeth due to thinner buccal bone and higher susceptibility to pressure. Molars exhibited milder changes, consistent with posterior teeth experiencing less severe resorption in subgroup analyses across studies [15,16]. Sahoo et al. [17] reported a reduction in the size of periapical lesions in ETT after fixed orthodontic treatment.

Multivariate regression identified distinct predictors: treatment duration was positively associated with resorption in both groups (stronger in vital teeth, B=0.13, p=0.001), likely due to cumulative force exposure amplifying inflammatory processes in vital pulps [18]. Greater pre-treatment root length was protective only in vital teeth (B=-0.4, p=0.001), possibly by distributing forces over a larger periodontal surface area and reducing the localized pressure. In the ETT group, mechanical factors (duration) predominated, with limited biological modulation, reinforcing that pulp vitality drives differential responses. These predictors align with established risk factors, including treatment duration and mechanics, highlighting pulp status as a key modifier [18]. Iglesias-Linares et al. [19] investigated the influence of interleukin-1β (IL-1β) gene polymorphisms on post-orthodontic EARR in ETT. Their findings suggest that genetic variations in the IL-1β gene may predispose ETT to varying degrees of EARR during orthodontic treatment, which differs from the response in vital pulp teeth.

The clinical implications of this study are substantial. OTM of highly obturated ETT appears safer regarding EARR than vital teeth, particularly for anterior retraction or intrusion, where vital teeth are at a higher risk. Clinicians should prioritize excellent endodontics before initiating orthodontics in cases with anticipated pulp compromise, as poor obturation was excluded here and may alter the outcomes. Minimizing treatment duration, especially for vital anterior teeth, and using lighter, controlled force can further mitigate risks. Routine radiographic monitoring (periapical films and CBCT, where indicated) is essential for early detection, with prompt force adjustment if resorption progresses beyond mild levels. The protective effect supports the inclusion of well-treated ETT in comprehensive plans without excessive concern, although periapical health surveillance remains prudent for detecting rare inflammatory or ankylotic complications.

This study has certain limitations. Its retrospective observational design precludes causal inference, and the findings should be interpreted as associations rather than effects. Although a split-mouth design and standardized institutional protocols were used, unmeasured factors such as precise force magnitude, force direction, clinician-dependent variations, and genetic susceptibility to external apical root resorption could not be fully accounted for, potentially resulting in residual confounding. In addition, restriction of the sample to patients aged 18-30 years improves internal validity by excluding growth-related influences but may limit the generalizability of the findings to adolescent orthodontic populations. Prospective multicenter studies employing standardized CBCT protocols, controlled biomechanics, and extended follow-ups are recommended to validate and refine these observations.

## Conclusions

This retrospective cohort study demonstrated that well-obturated ETT exhibited significantly less EARR during OTM than vital teeth. The absence of vital pulp tissue appears to confer a protective effect against inflammatory resorption, with ETT showing predominantly mild or no resorption, whereas vital teeth were more susceptible to moderate and severe degrees. Treatment duration emerged as a key risk factor in both groups, whereas a longer pre-treatment root length was protective only in vital teeth. These findings support the safe orthodontic movement of high-quality ETT, with emphasis on excellent endodontic treatment, controlled mechanics, and regular radiographic monitoring to optimize long-term outcomes and minimize iatrogenic risk.
